# Design, Manufacturing, and Educational Evaluation of a Low-Cost Venipuncture Training Simulator

**DOI:** 10.7759/cureus.106831

**Published:** 2026-04-11

**Authors:** Stefan Tserovski, Konstantinos Papadakis, Krasimir K Yanev, Rene D Mileva-Popova, Todor G Bogdanov

**Affiliations:** 1 Orthopedics and Traumatology, Medical University - Sofia, Sofia, BGR; 2 Physiology and Pathophysiology, Medical University - Sofia, Sofia, BGR; 3 Dermatology and Venereology, Medical University - Sofia, Sofia, BGR; 4 Medical Physics, Medical University - Sofia, Sofia, BGR

**Keywords:** clinical skills, in-house production, medical education, medical students, venipuncture

## Abstract

Venipuncture is a fundamental clinical skill that requires repeated practice in a safe learning environment before being performed on patients. However, commercial venipuncture simulators are often expensive and not always available in sufficient numbers for repeated student training. This study aimed to design, manufacture, and evaluate a low-cost, reusable venipuncture training model for medical education. The simulator was developed using a silicone-based soft-tissue model with embedded silicone tubing simulating superficial veins at different depths. A passive hydrostatic pressure system was used to provide visual feedback during successful venipuncture. The model was manufactured using a 3D-printed mold, platinum-cure silicone, silicone tubing, and a porous support layer to improve durability and fluid absorption. The total material cost per model was approximately 4-5 EUR. The model was used in a practical training session with 50 medical students as part of a scheduled educational activity. Following the training, participants were invited to complete a voluntary and anonymous questionnaire designed to evaluate the perceived realism, usability, and educational value of the model. The questionnaire consisted of 10 items rated on a 5-point Likert scale. The instrument was developed specifically for this study and was not previously validated; therefore, the results should be interpreted as exploratory measures of user perception. Students reported high satisfaction across all evaluated categories, particularly ease of use, the ability to perform repeated venipuncture, and the visual feedback system. This low-cost, in-house manufactured venipuncture simulator provides a practical and sustainable solution for procedural training. Its affordability, reusability, and ease of manufacturing make it suitable for widespread use in medical education, particularly in settings with limited access to commercial simulation equipment.

## Introduction

Venipuncture is a common medical procedure that involves inserting a needle into a vein, typically in the arm, to draw blood or administer fluids and medications. It is one of the most frequently performed invasive procedures in clinical practice and represents a core competency for medical students and healthcare professionals. Despite its routine nature, venipuncture requires the development of fine motor skills, proper needle angle control, depth perception, and tactile feedback recognition. For example, an incorrect needle angle may result in missing the vein, while excessive insertion depth may lead to vessel perforation or patient discomfort. These challenges highlight the importance of repeated, supervised practice in a safe learning environment. However, traditional training based solely on supervised practice with real patients is increasingly constrained by ethical considerations, patient safety concerns, reduced patient availability, and a growing number of medical students. For these reasons, simulation-based training has become an essential component of modern medical education [1,2].

Simulation-based medical education allows learners to acquire and practice procedural skills in a controlled, risk-free environment, where mistakes can be made without harming patients. Simulation provides standardized training conditions, immediate feedback, and the possibility for repeated practice, all of which are essential for the acquisition of psychomotor skills and procedural competence [3,4]. A growing body of literature, including systematic reviews, meta-analyses, and observational studies, suggests that simulation-based training is associated with improvements in technical skills, learner confidence, and clinical performance compared to traditional clinical training alone, although the strength of evidence varies across study designs. Higher-level evidence, such as meta-analyses, provides stronger support, while individual observational studies should be interpreted with appropriate caution [5,6]. Furthermore, simulation-based education has been associated not only with improved procedural performance in simulated settings but also with improved performance in real clinical environments and improved patient safety outcomes [6].

An important educational concept underlying simulation-based training is deliberate practice, which involves repeated performance of a task, immediate feedback, and gradual improvement through structured training. Deliberate practice is a key factor in acquiring expert-level performance in procedural and clinical skills [2,7]. In this context, simulation models that allow repeated practice without model deterioration are particularly valuable in medical education.

Although high-fidelity commercial simulators are widely used for procedural training, they are often expensive and not always available in sufficient numbers for large student groups. This limits the opportunity for repeated hands-on practice, which is essential for skill acquisition. As a result, there is increasing interest in low-cost, locally manufactured simulation models that can be produced in larger quantities and adapted to specific educational needs [3,8]. In-house manufacturing of task trainers enables design flexibility, rapid prototyping, easy modification, and significant cost reduction, making simulation-based training more accessible and scalable.

Venipuncture training models belong to the category of part-task trainers, which are designed to simulate a specific anatomical region or procedural skill. The educational value of part-task trainers lies in their ability to provide focused training on specific psychomotor skills, such as needle insertion, depth control, and hand-eye coordination, without the complexity of full-body simulators [4,9]. For venipuncture training in particular, important design characteristics include realistic tissue resistance, palpable vessel structures, simulated blood flashback, durability, and the ability to withstand multiple punctures.

This technical report describes the design, manufacturing process, and educational evaluation of a low-cost, reusable venipuncture training simulator developed with silicone materials, embedded tubing, and a passive fluid system. The model was designed to be affordable, easy to manufacture, leak-resistant, and suitable for repeated use in student training sessions. In addition to the technical description of the model, this study presents the results of a student satisfaction evaluation conducted after practical training with the simulator.

## Technical report

Design concept and functional requirements

**Figure 1:**
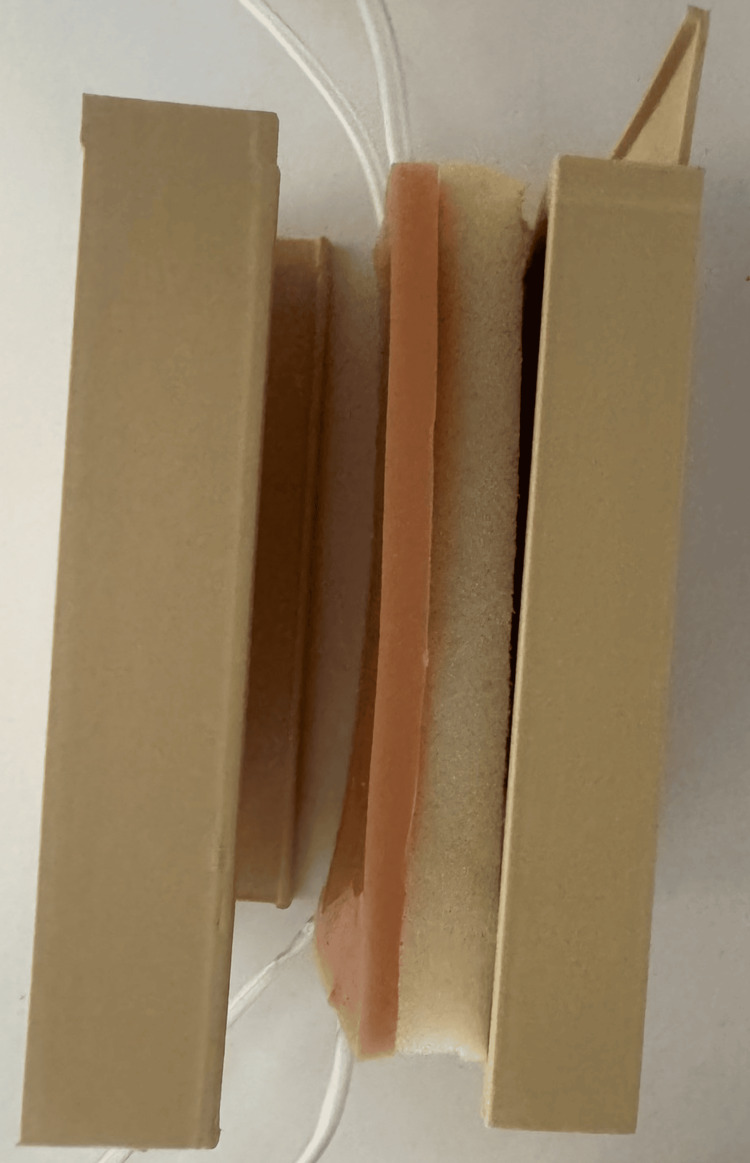
Structural design of the low-cost venipuncture training model. The model consists of a silicone tissue-mimicking layer with embedded silicone tubing representing veins at different depths (approximately 2 mm and 4 mm), a porous support layer providing structural resistance and fluid absorption, and a rigid 3D-printed holder. The tubing is connected to an external fluid reservoir to simulate blood flashback.

The venipuncture training simulator was developed as a low-cost, reusable, and easily reproducible part-task trainer intended for repetitive practice of peripheral venipuncture in a controlled educational environment. The primary design objectives included anatomical plausibility, realistic tactile feedback, durability under repeated punctures, ease of manufacturing, and minimal material cost. These design principles are consistent with current recommendations for part-task trainers used in procedural skills education, where focused psychomotor training and repetitive practice are essential for skill acquisition [4,8]. The simulator consists of a silicone tissue layer, embedded silicone tubing simulating veins, a porous support layer, and a rigid external holder. The structural design of the model is shown in Figure 1. The model consists of a silicone tissue layer with embedded silicone tubing simulating veins, a porous support layer, and a 3D-printed holder. The tubing is connected to a fluid reservoir to simulate blood flashback.

The model was designed to simulate superficial peripheral veins embedded within soft tissue. The simulator consists of a silicone tissue-mimicking layer, embedded silicone tubing representing veins, a porous support layer providing structural resistance, and a rigid external holder produced by 3D printing. The design allows repeated venipuncture attempts without significant structural degradation of the model.

A key functional requirement was the ability to provide immediate visual feedback upon successful venipuncture. For this purpose, a passive fluid system was incorporated into the model, enabling simulated blood flashback when the needle enters the tubing lumen. Immediate feedback is considered an important component of simulation-based training, as it improves skill acquisition and procedural accuracy during repeated practice [2,7].

Another important design requirement was durability. Commercial venipuncture models often degrade after multiple punctures due to leakage from artificial vessels and tearing of the surrounding material. To address this limitation, silicone tubing was used instead of cast channels, reducing the risk of leakage after repeated needle insertions. In addition, a porous support layer was placed beneath the silicone tissue layer to absorb small amounts of fluid in the event of minor leakage, thereby extending the model's usable life.

The model was also designed to allow variation in vein depth and vessel diameter during manufacturing, enabling the creation of models with different levels of difficulty. This allows the simulator to be used for both beginner and more advanced learners, which is an important characteristic of effective simulation-based training systems [3].

Structural design and geometry

The simulator includes two embedded silicone tubes representing superficial veins, positioned at different depths beneath the silicone tissue layer. The tubes have an outer diameter of approximately 2-3 mm and an inner diameter of approximately 1.5-2 mm, corresponding to the dimensions of peripheral veins commonly used for venipuncture. The tubes are positioned at depths of approximately 2 mm and 4 mm below the surface, respectively, allowing trainees to experience varying levels of puncture difficulty and the needle angulation required.

The presence of two vessels at different depths within a single model allows repeated practice and comparison between easier and more difficult puncture scenarios. This variability is important for procedural training, as skill acquisition improves when learners are exposed to different levels of difficulty rather than a single fixed scenario [7].

The silicone tissue layer thickness was selected to provide realistic resistance during needle insertion while still allowing palpation of the simulated vessels. The rigid outer holder maintains the model's shape and allows for easy handling during training sessions.

Materials

The soft tissue component of the model was fabricated using a platinum-cure silicone elastomer (EcoFlex 00-30), selected for its low Shore hardness and elastic properties that approximate those of human soft tissue. Silicone materials are commonly used in medical simulation models due to their durability, elasticity, and resistance to repeated puncture [4].

The simulated vessels were made from silicone tubing, which offers greater resistance to leakage than cast silicone channels. The tubing system allows continuous fluid flow and repeated puncture without significant damage to the vessel walls.

A porous polyurethane foam layer was placed beneath the silicone tissue layer to provide structural support and absorb fluid in case of minor leakage. This layer also contributes to tactile feedback during needle insertion by providing resistance similar to that of subcutaneous tissue. The main technical characteristics of the model are summarized in Table 1.

**Table 1:** Technical characteristics of the venipuncture training model.

Parameter	Description
Tissue material	Platinum-cure silicone (EcoFlex 00-30)
Simulated veins	Silicone tubing
Vein diameter	2-3 mm
Vein depth	2 mm and 4 mm
Support layer	Polyurethane foam
Holder	3D-printed plastic
Fluid system	Passive hydrostatic system
Flashback	Yes
Reusability	Yes
Cost per model	4-5 EUR

The external holder and mold were produced using fused deposition modeling (FDM) 3D-printing technology. The use of 3D printing enables rapid prototyping, easy design modification, and batch production of multiple models simultaneously, which is an important advantage in simulation-based education, where multiple identical training units are required [3,8].

Manufacturing process

The manufacturing process begins with producing a mold using 3D printing. The mold includes predefined channels and positioning elements to ensure accurate placement of the silicone tubing at the desired depths.

During fabrication, the silicone tubes are positioned inside the mold and fixed in place. Liquid silicone is then poured into the mold to form the tissue layer. The curing time for the silicone is approximately 24 hours at room temperature, according to the manufacturer's specifications. After curing, the silicone block containing the embedded tubing is removed from the mold and placed into the rigid external holder together with the porous support layer.

Multiple models can be produced simultaneously using the same mold design, enabling batch production and reducing per-unit manufacturing time. The manufacturing process does not require specialized industrial equipment and can be performed in a laboratory or university workshop environment, making the model suitable for in-house production.

Fluid system and simulated flashback

**Figure 2:**
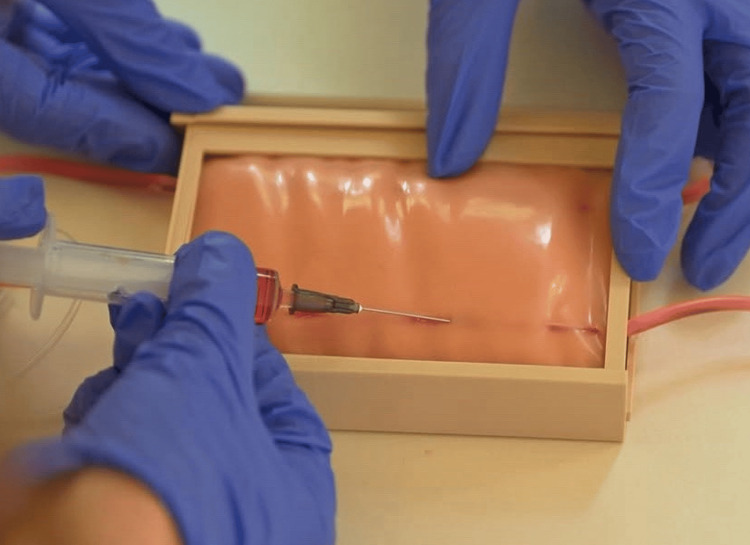
Passive fluid system for simulated blood flashback. The system includes silicone tubing connected to an external fluid reservoir positioned above the model to generate hydrostatic pressure. When the needle enters the tubing lumen, fluid flows back into the needle hub, simulating clinical flashback.

The model incorporates a passive fluid system designed to simulate blood flashback during successful venipuncture. The system consists of silicone tubing connected to an external fluid reservoir filled with colored water. The reservoir is positioned approximately 80-100 cm above the model to create hydrostatic pressure within the system. The passive fluid system used to simulate blood flashback is presented in Figure 2. The external fluid reservoir is positioned above the model to generate hydrostatic pressure, allowing immediate visual flashback when the needle enters the simulated vein.

When the needle enters the lumen of the silicone tube, hydrostatic pressure immediately causes the colored fluid to backflow into the needle hub, simulating the clinical flashback observed during successful venipuncture. This system does not require pumps or electrical components, which simplifies the design and reduces cost while still providing effective training feedback. Immediate visual feedback is an important component of procedural learning, as it allows trainees to recognize successful needle placement and adjust their technique accordingly during repeated attempts [2,7].

Cost and reusability

One of the primary design goals of the model was low production cost. The total material cost for a single model is approximately 4-5 EUR, including silicone material, silicone tubing, porous support material, and 3D-printing material for the holder and mold. The use of reusable molds and holders further reduces the cost per unit when multiple models are produced.

The model is designed for repeated use during multiple training sessions. The use of silicone tubing instead of cast vessels significantly reduces leakage and structural damage after repeated punctures. In addition, the porous support layer absorbs small amounts of fluid, preventing external leakage and maintaining the model's functionality over time.

The combination of low cost, durability, and ease of manufacturing makes this model particularly suitable for training large groups of medical students, where access to commercial simulators may be limited due to cost constraints. Low-cost training models enable repeated practice, a key factor in procedural skill acquisition and confidence development among medical students [2,7].

## Discussion

Simulation-based training has become an essential component of modern medical education, particularly for procedural skills that require psychomotor coordination, tactile feedback, and repeated practice. Traditional apprenticeship-based models of teaching, often summarized as “see one, do one, teach one,” are increasingly insufficient due to reduced patient availability, increased student numbers, and growing emphasis on patient safety and ethical considerations in medical training [10,11,12]. For invasive procedures such as venipuncture, simulation provides a safe environment in which students can develop technical skills before performing procedures on real patients [1,5].

A key concept in procedural skills training is deliberate practice, which involves repetitive performance of a task with immediate feedback and progressive improvement in performance. Deliberate practice has been shown to be one of the most important factors in the acquisition of expert-level performance in procedural skills [7,13]. Simulation models are particularly suitable for deliberate practice because they allow repeated attempts without risk to patients and without the time constraints of clinical environments [3,4]. Therefore, the availability of durable and reusable training models is of critical importance in medical education.

Commercial venipuncture simulators are widely used in medical skills laboratories; however, their high cost often limits the number of available training units. This restricts the amount of hands-on practice each student can perform, which may negatively affect skill acquisition. In contrast, low-cost, locally manufactured models allow the production of multiple units, enabling simultaneous practice by larger groups of students and increasing the total number of performed procedures during training sessions. The ability to perform repeated procedures is a major factor in procedural skill acquisition and confidence development [2,7].

The venipuncture model presented in this study was designed with a focus on low cost, durability, and functional realism. Several design features distinguish this model from many basic training models. First, the use of silicone tubing instead of molded channels significantly reduces leakage after repeated punctures and increases the lifespan of the model. Second, the addition of a porous support layer beneath the silicone tissue layer helps absorb small amounts of fluid and improves tactile feedback during needle insertion. Third, the passive hydrostatic fluid system provides immediate visual feedback in the form of simulated blood flashback, which is an important component of procedural learning. Immediate feedback allows students to recognize successful needle placement and adjust their technique during repeated attempts, which improves learning efficiency during simulation-based training [3,13].

Another important advantage of the model is the possibility to vary vein depth and tubing diameter during manufacturing. This allows the creation of models with different difficulty levels, which is important for progressive skills training. Beginners can start with larger and more superficial vessels, while more advanced learners can practice on smaller or deeper vessels. Progressive difficulty is considered an important component of effective simulation-based education and deliberate practice training models [7].

From an educational perspective, this model belongs to the category of part-task trainers, which are specifically designed to teach a single procedural skill. Part-task trainers are widely used in medical education because they allow focused, repetitive training of specific psychomotor skills without the complexity of full-scale simulators [4,10]. For venipuncture training in particular, the most important characteristics of a training model include realistic tissue resistance, vessel palpability, flashback simulation, durability, and the ability to withstand multiple punctures. The model described in this report was designed to address these specific requirements. This study addresses a practical gap related to the accessibility and scalability of simulation-based training, rather than providing high-level evidence of educational effectiveness.

Educational evaluation

The educational evaluation was conducted as an exploratory, single-session assessment during a scheduled practical training session. All attending medical students (n = 50) were invited to participate. The questionnaire was administered immediately after the training session in paper form. All attending students completed the questionnaire (response rate: 100%), and no missing data were recorded. Following the training, students completed a voluntary and anonymous questionnaire consisting of 10 items rated on a 5-point Likert scale, assessing perceived realism, usability, and educational value. The questionnaire was developed specifically for this study and was not a previously validated instrument; therefore, the results should be interpreted as exploratory measures of user perception. The educational evaluation conducted in this study demonstrated high levels of student satisfaction across all evaluated categories, including realism, usability, and educational value. The highest-rated aspects of the model were ease of use, the ability to perform multiple punctures, and the visual flashback system. These findings are consistent with the principles of simulation-based education, where repeated practice and immediate feedback are considered key factors in skill acquisition [2,3]. Descriptive statistical analysis was performed using Microsoft Excel (Microsoft Corp., Redmond, WA), and results are presented as mean values with standard deviations. The results of the student satisfaction questionnaire are presented in Table 2.

**Table 2:** Student satisfaction questionnaire results (n = 50).

No.	Statement	Mean ± SD
1	The model realistically simulates human soft tissue	4.32 ± 0.65
2	The model realistically simulates venous puncture	4.41 ± 0.58
3	The resistance during needle insertion felt realistic	4.18 ± 0.72
4	The model was easy to use	4.74 ± 0.44
5	The model helped me understand needle angle and depth	4.52 ± 0.51
6	The flashback was useful	4.68 ± 0.47
7	The model improved my confidence	4.36 ± 0.63
8	Repeated punctures were useful	4.81 ± 0.39
9	Suitable for student training	4.77 ± 0.42
10	I would recommend this model	4.83 ± 0.38

**Figure 3:**
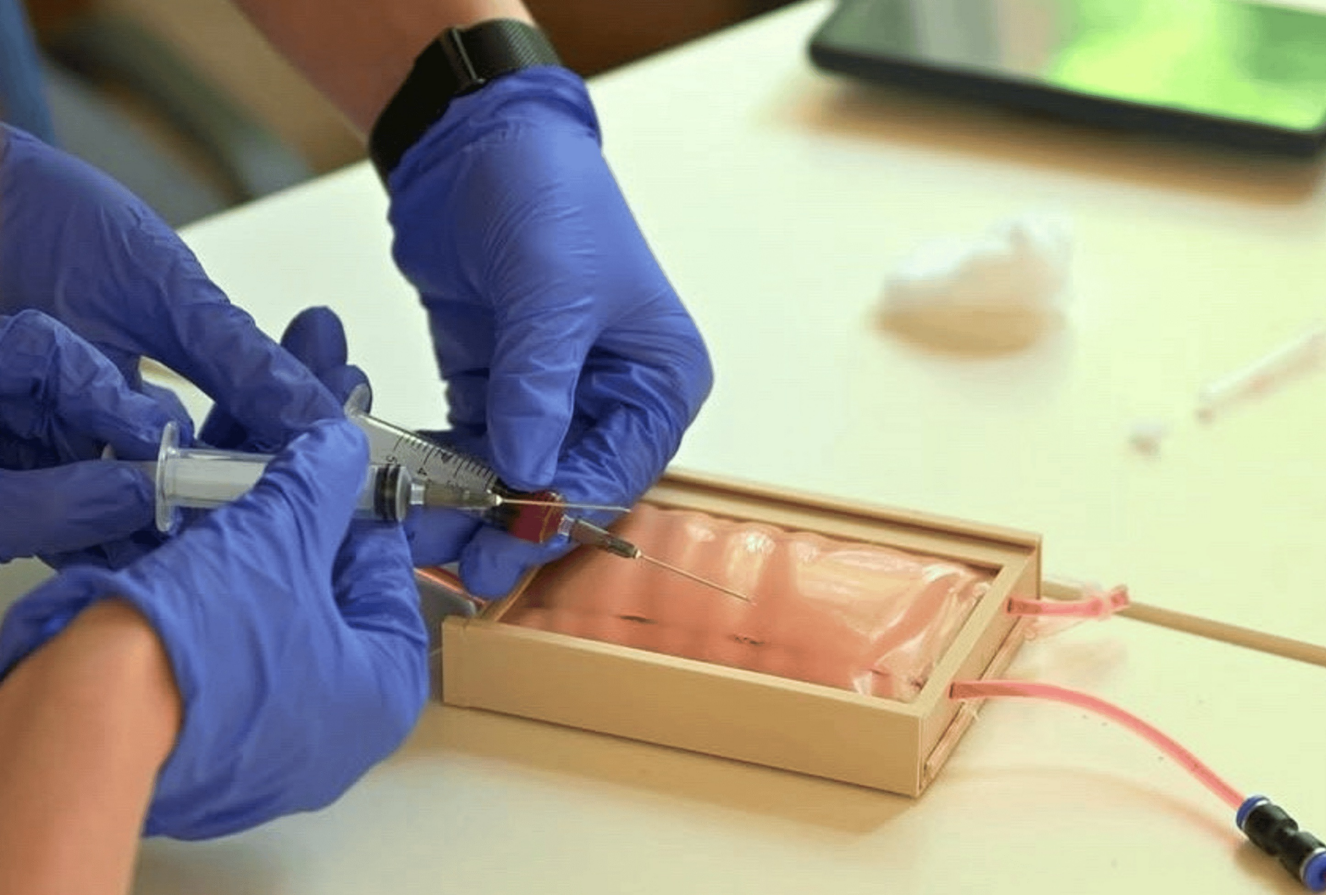
Venipuncture training using the developed simulator. The image shows repeated venipuncture attempts performed by medical students under controlled training conditions, with immediate visual feedback provided by the simulated flashback system.

Mean values above 4.0 indicate a high level of perceived agreement with the evaluated statements, suggesting positive user perception of the model’s usability and training relevance. These results indicate high perceived usability and training relevance of the model, although they reflect subjective student evaluation rather than objectively measured procedural performance. Interestingly, the realism of tissue resistance received slightly lower scores compared to usability and educational value, although it was still rated positively. This finding is consistent with previous studies showing that, for beginner learners, the opportunity for repeated practice and immediate feedback may be more important than perfect anatomical realism [3,4]. In early stages of skill acquisition, functional realism and the ability to practice repeatedly are often more important than high-fidelity simulation. The use of the model during student training is shown in Figure 3. The model allows repeated venipuncture attempts under controlled training conditions with immediate visual feedback upon successful vessel puncture.

Students also reported that the model helped improve their confidence in performing venipuncture. Confidence development is an important outcome of simulation-based training, as a lack of confidence is a known barrier to performing procedures in clinical settings. Previous studies have shown that simulation-based training improves both procedural performance and learner confidence, particularly when students are allowed to perform multiple repetitions of the procedure in a controlled environment [5,6].

An important advantage of the presented model is its durability and resistance to leakage, which allows a large number of punctures to be performed without significant damage to the model. This makes the model suitable for repeated use in training sessions with large student groups. From an economic perspective, the low production cost allows multiple models to be produced, which increases student access to hands-on training. Increased access to training opportunities is directly related to improved procedural competence in medical students [2,7]. No inferential statistical analysis was performed, as the evaluation was designed as an exploratory assessment of user perception rather than a hypothesis-driven study.

This study has several limitations. First, the educational evaluation was based on student satisfaction and perceived educational value rather than objective assessment of procedural performance. No pre-training and post-training measurements were performed, and no objective indicators such as venipuncture success rate, number of attempts, procedure time, or needle insertion accuracy were assessed. Therefore, the present findings cannot be interpreted as evidence that the model improves procedural competence. Second, the evaluation was conducted during a single training session, and long-term skill retention was not assessed. Third, all students were trained using the developed simulator, without a comparison group using either a commercial venipuncture trainer or another training approach. As a result, the present study does not allow conclusions regarding comparative educational effectiveness.

Fourth, the questionnaire used in this study was developed for the purposes of the training session and was not a previously validated instrument. Consequently, the questionnaire results should be interpreted as exploratory measures of user perception rather than as validated measures of realism or educational impact. In addition, the data analysis was descriptive and was limited to mean values and standard deviations. The study also has limitations related to implementation and reproducibility. Although the manufacturing process and materials are described in detail, some technical parameters may require further standardization for exact replication in other settings. In addition, durability and leakage resistance were assessed during repeated practical use, but were not quantified in a formal mechanical testing protocol.

Finally, because the simulator was developed and evaluated by the same academic team, the possibility of positive-response bias cannot be fully excluded, even though the student questionnaire was completed anonymously. Future studies should include objective performance metrics, pre- and post-training assessment, comparison with commercial simulators or alternative training methods, validated evaluation tools, and formal durability testing. Within these limitations, the present work should be interpreted primarily as a technical report describing the development and feasibility of a low-cost venipuncture training model, together with preliminary user feedback.

## Conclusions

The venipuncture training simulator described in this technical report was developed as a low-cost, reusable, and easily reproducible model for procedural training in medical education. The model combines a silicone soft-tissue structure, embedded silicone tubing simulating veins, a porous support layer for fluid absorption, and a passive hydrostatic system that provides immediate visual flashback upon successful venipuncture. Its main practical advantages include low material cost, ease of manufacturing, reusability, and suitability for repeated training of large student groups.

The educational evaluation showed high student satisfaction regarding usability, repeated puncture capability, and immediate visual feedback, with mean scores exceeding 4.0 across all evaluated items. However, because the evaluation was based on subjective questionnaire data obtained after a single training session, the present study does not provide objective evidence of improved procedural performance or long-term skill retention. Therefore, the model should be regarded as a feasible and accessible training tool with promising educational potential, rather than as a fully validated educational intervention. The positive user feedback suggests that the model may support repeated practice and confidence development in venipuncture training, although this was not objectively measured in the present study. Further studies should include objective skill assessment, comparative evaluation, and long-term follow-up.
